# Nanoscale and Tensile-Like Properties by an Instrumented Indentation Test on PBF-LB SS 316L Steel

**DOI:** 10.3390/ma17010255

**Published:** 2024-01-03

**Authors:** Giovanni Maizza, Faisal Hafeez, Alessandra Varone, Roberto Montanari

**Affiliations:** 1 Department of Applied Science and Technology, Politecnico di Torino, 10129 Torino, Italy; 2 Department of Industrial Engineering, University of Rome “Tor Vergata”, 00133 Roma, Italy; faisal.hafeez@students.uniroma2.eu (F.H.); alessandra.varone@uniroma2.it (A.V.); roberto.montanari@uniroma2.it (R.M.)

**Keywords:** selective laser melting (PBF-LB), instrumented indentation test (IIT), nano and macro, SS 316L steel, remnant residual stress

## Abstract

The mechanical properties of a defect-free laser melting (PBF-LB) deposit of an AISI 316L steel alloy were assessed by means of an instrumented indentation test (IIT), at both the macro- and nano-scales. The inherent non-equilibrium microstructure of the alloy was chemically homogenous and consisted of equiaxed grains and large-elongated grains (under the optical microscope) with irregular outlines composed of a much finer internal cell structure (under the scanning electron microscope). Berkovich and Vickers indenters were used to assess the indentation properties across individual grains (nano) and over multiple grains (macro), respectively. The nano-indentation over the X-Y plane revealed nearly constant indentation modulus across an individual grain but variable on average within different grains whose value depended on the relative orientation of the individual grain. The macro-indentation test was conducted to analyze the tensile-like properties of the polycrystalline SS 316L alloy over the X-Y and Y-Z planes. The macro-indentation test provided a reliable estimate of the ultimate tensile strength (UTS-like) of the alloy. Other indentation properties gave inconsistent results, and a post factum analysis was, therefore, conducted, by means of a new approach, to account for the presence of residual stresses. The already existing indentation data were supplemented with new repeated indentation tests to conduct a detailed analysis of the relaxation ability of compressive and tensile residual stresses. The developed methodology allows the effect of residual stresses and the reliability of measured macro-indentation properties to be examined as a function of a small group of indentation parameters.

## 1. Introduction

Selective laser melting (PBF-LB) is a laser-based additive manufacturing technology that is expanding in industry to fabricate new complex products as well as to repair existing parts that have been damaged during service operation [1]. PBF-LB is a continuous process of melting and depositing a powder feedstock, layer by layer, using a laser heat source. PBF-LB is becoming more and more accepted because of its eco-friendly features, in terms of material utilization and low waste, compared to conventional manufacturing [2]. PBF-LB has the capability of producing any part that can be 3D modeled. PBF-LB built can be customized to deposit materials similar or dissimilar to the substrate [3]. Austenitic stainless steels, such as SS 316L, are of particular interest for applications in challenging high corrosive environments [4], as they maintain an excellent combination of high strength and ductility, even at low temperatures [5]. Considering engineering applications, SS 316L exhibits obvious intergranular corrosion resistance to most chemicals, salts and acids, and Molybdenum (Mo) provides better protection in marine environments [6]. SS 316L steel combines outstanding strength, corrosion resistance, biocompatibility properties [7,8] and low-temperature fracture toughness. When PBF-LB is combined with SS 316L for the fabrication of parts, a characteristic microstructure, composed of fine equiaxed and columnar grains, with a cellular substructure, is produced because of the different deposition and cooling rate conditions. Bertoli et al. [9] observed the cellular substructure in a variety of SS 316L components, fabricated by means of PBF-LB, using 60, 175, and 380 W of laser power and 30, 750, and 2500 mm/s of scanning speed, with the largest cell size (839 ± 125) at 60 W, 30 mm/s, and the smallest (478 ± 76) at 380 W, 2500 mm/s PBF-LB settings. The measured UTS of the three samples was 633 ± 2 MPa for the lowest laser power settings, 619 ± 24 MPa for the medium ones, and 529 ± 18 MPa for the highest power settings. In another study, Birnbaum et al. [10] reported that in PBF-LB build SS 316L samples the appearance of this typical substructure depended on the cooling rate during the advancement of the solid/liquid interface. In the literature, the most common method used to test the mechanical performance of an PBF-LB part is the tensile test, although chemical and microstructure gradients along the growth direction may introduce accidental non-uniform elastic and plastic deformations. A more versatile method to assess the mechanical properties, which also accounts for any eventual microstructural anisotropy, is the instrumented indentation test (IIT). The materials tested in such a method can be in the form of free-standing thin films or multilayers, coatings, or bulk solids [11,12,13]. This method permits different scales of the microstructure of the material to be characterized using differently shaped indenters. ISO standard 14577-1 outlines three different scales for IIT:

nano-scale: h_max_ ≤ 0.2 µm
micro-scale: F_max_ < 2 N, h_max_ > 0.2 µm
macro-scale: 2 N ≤ F ≤ 30 kN

The test is particularly useful in that it records the load and depth at any instant of time. During IIT, the indenter is forced to penetrate the tested material up to a defined peak load (F_max_) under a controlled (loading or penetration) rate, then held at the peak load for a specific time (t_holding_), and finally, released at a controlled rate [14].

Upon indentation, the material is deformed elasto-plastically, while plasticity tends to be fully developed (Tabor’s requirement), which is necessary to measure indentation hardness. Indentation properties other than indentation hardness (H_it_), and indentation modulus (E_it_) are also extracted from the recorded load-depth data. Although nano-indentation (nIIT) can sense the mechanical response of the material near the surface or in the shallower subsurface, macro-indentation (MIIT) is able to sense the material response across a larger bulk volume. A large, indented volume likely contains most of the essential features of the microstructure that resemble those involved in the tensile or compression test. Thus, macro-indentation may provide an equivalent testing method to assess the elastoplastic response of a material in a quick manner. Nanoscale artefacts, such as surface asperities [15], indenter tip blunting [16], superficial oxide layers [17], or indentation size effects (ISE) [18], may affect the indentation properties, thus making them difficult to compare. However, MIIT can mitigate all the above mentioned detrimental superficial effects, especially the ISE effect [19]. 

To date, most of the researchers working on IIT have focused on nanoindentation to investigate the mechanical properties of ceramics, polymers, and metallic materials. In metallic materials, maximum nanoscale depth can reach up to 0.2 microns, which is beneficial for assessing the mechanical properties of thin films and coatings [20,21,22]. On the other hand, nIIT has been successfully utilized, with specific reference to SS 316L, to inspect the effects of surface modifications, such as those induced by irradiation [23], pulsed plasma [24], nitriding [25], sol-gel thin coating [26], and more. To the best of the authors’ knowledge, no studies, except for the one published by [27] on the fatigue behavior of PBF-LB SS 316L under different orientations (vertically, horizontally and at a 45° angle) using Vickers instrumented macro-indentation, have been conducted on the elastoplastic behavior of either SS 316L steel or its PBF-LB counterpart. These authors complemented their results with a standard Vickers hardness (HV_30_) test, which they found was highest in the vertically printed direction (229 ± 3) but in contrast to the lowest tensile strength (600 ± 1 MPa). Since our primary goal has been to explore the plastic behavior of PBF-LB SS 316L toward its ultimate tensile strength (UTS), to find a direct relationship with the standard Vickers hardness, we have avoided referring to existing studies based on spherical indentation testing as these would be more concerned with the elastoplastic behavior of the material near its yield strength.

A unique advantage of MIIT over nIIT Is that samples are less sensitive to finishing methods and roughness levels, and the tested surface is just marginally affected (nm or µm scale). Various metallic materials have been investigated using Vickers MIIT to measure the elastic modulus of a hardness-block made of martensitic stainless steel [28], the yield stress, and the work hardening exponent of 316L and 420 steel by implementing FEM and a genetic algorithm on the P-h data [29], the indentation modulus and creep of a stainless steel reference block, Al alloy, copper alloy, Cu-Cr-Zr alloy [30], and more. Although MIIT is not effected by the ISE, there are still several factors that can influence the measured properties to a great extent, such as the Vickers indenter tip blunting effect on the γ-TiAl alloy [31], FEM-based research on the effect of surface roughness on the macro-spherical indentation P-h curve of SS 316L [32], and investigating residual stresses with Vickers macro-indentation on HY80 and HY100 steel welds [33]. 

However, too large peak loads might result in excessive damage for highly finished engineering products, due to large imprints. Thus, a trade-off load value should be selected to minimize surface damage while avoiding ISE effects, and to provide a macroscopic material response that is equivalent to the tensile/compression test. 

Although nIIT and MIIT have been individually and independently investigated in various studies, both have rarely been combined to share their relative benefits, for instance [20,21,22]. A dual-scale correlation is more suitable for building prototypes that are virtually free from common defects (e.g., pores, oxides, inclusions, lack of fusion), as in the case considered here. Therefore, a systematic investigation of both scales on an PBF-LB SS 316L sample has been beneficial to help cross-link material properties at different length scales, i.e., over the indent regions of several grains and within individual grains. The provision of the tensile-like properties of PBF-LB SS 316L offers a useful baseline for benchmarking different scanning strategies or manufacturing processes. MIIT, even though much less common than nIIT, is of paramount importance for industry and engineering as tensile properties play a leading role in most national and international engineering design standards. Moreover, nIIT and MIIT can be used during and after PBF-LB, to accelerate the process setup and to assess the structural performance and quality of the final products, respectively. For the current study, the PBF-LB fabricated block was supplied by an PBF-LB device manufacturer after extensive research devoted to finding an optimal PBF-LB process window to produce advanced cost-effective SS 316L parts for use in the biomedical and in the transportation industry. 

The paper is organized in three sections, namely, Material and Methods, Results, and Discussion. The latter section, in turn, has been divided into two subsections: Section 4.1 first discusses the relationships emerging between nano- and macro-indentation properties and ends with recognizing the lack of useful means to elucidate the anomalies and inconsistencies observed among the indentation properties; Section 4.2, after briefly introducing the topic of residual stresses in the contest of IIT, re-examines the possible causes of anomalies with a new perspective to account for the breakdown of the fundamental condition of full elastic contact between the indenter and the investigated material upon unloading (ISO 14577-1,2 [14,34]). A new approach has been illustrated to interpret the IIT phenomena concurrently affected by residual stresses. A criterion to discern whether or not a measured indentation property has been impaired by residual stress is also presented. 

## 2. Materials and Methods

**Figure 1:**
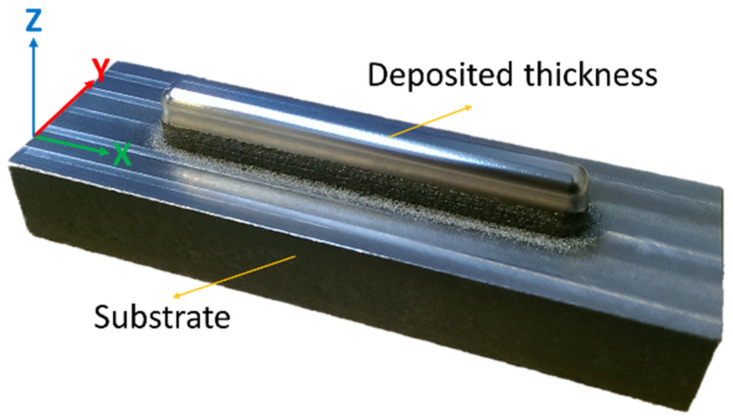
PBF-LB SS 316L steel deposit on an AISI 1020 steel substrate.

**Figure 2:**
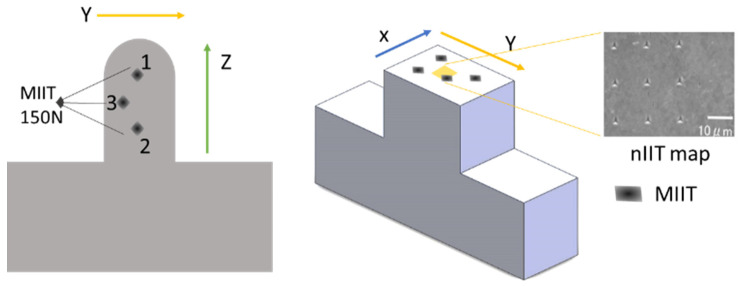
Schematic of the locations of n.3 MIIT on the Y-Z and X-Y planes (dark diamonds); the inset shows the n.9 nIIT (3 × 3 matrix in the 26 × 26 µm^2^ square in yellow) locations over the X-Y plane.

The prototype was built using a pre-alloyed high flowable (LPW-316-AABE) powder of an average size of 44–88 µm. The laser power was set to 350 W, and the scanning rate was set to 300 mm/min. The deposit (5 × 7.5 × 65 mm^3^) was fabricated, layer-by-layer (Figure 1), along the Z-axis using a scanning strategy consisting of repeated X-direction stripe layers superimposed onto a Y-direction stripe layer [35,36,37]. A solid block (24.3 × 7.6 × 70 mm^3^) of AISI 1020 steel was employed as a substrate. The first part of the deposit exhibited planar vertical walls of up to 5 mm in height (Figure 2). The surface of the top hemispherical cap (2.5 mm radius) was finished by grinding. The metallographic and indentation sample consisted of a transversal 5 mm thick slice (corresponding to the Y-Z plane in Figure 2), which was extracted from the mid-length of the entire deposit to minimize processing end effects. The inherent machining and polishing operations were performed with an abundant refrigerant flow and a very low feed rate to prevent local overheating and/or accidental alteration of the original PBF-LB microstructure. The sample was ground with 300 to 4000 grit SiC smearing paper, and finally mirror polished using 6-, 3-, and 1-micron diamond particle suspensions. The polished samples were etched with aqua regia for 65–75 s prior to the indentation testing.

The characteristic scale of the SS 316L steel microstructure was assessed using optical micrographs (DMI 3000 M, Leica, Wetzlar, Germany) and a scanning electron microscope (TESCANS 9000 G, Tescan group, Brno, Czech Republic). The microstructure was surveyed thoroughly by means of optical (by using 5× to 100× magnifying objective lenses) and scanning electron microscopy (1500× to 12,000× magnification and at 20 kV) to reveal the most relevant microstructure features at different locations of the build and to confirm the absence of porosity and other processing defects because of the found optimal process conditions. Indeed, the sample essentially appeared to be free of visible defects, except for some sporadic micro- or submicron-sized spherical pores. It is uncertain whether such pores were induced by PBF-LB or, and it seems more likely, from corrosion pitting caused during the search for an optimal etching condition. Additionally, EDS (energy-dispersive X-ray spectroscopy) and EBSD (electron backscatter diffraction) analyses were carried out to analyze the chemical homogeneity and to confirm the fully austenitic phase in the samples, respectively. The EDS and EBSD figures have, therefore, been omitted from this paper.

The dual-scale IIT (nIIT and MIIT) was used to sense the elastoplastic properties of an PBF-LB deposit with microstructure features spanning over different length scales [38]. To minimize any possible interference between the nIIT and MIIT stress-strain fields, the former was performed first, followed by MIIT. The nIIT was used to sense the elastoplastic properties at a typical individual grain scale, whereas MIIT was utilized to sense larger volumes containing several grains. The inherent nIIT aspects are from now on denoted with the *n* prefix to discern them from the MIIT aspects. 

Prior to nIIT (Hisytron TI 950 TriboIndenter, Berkovich indenter, Massachusetts, US) and MIIT (Zwick Röell ZHU 2.5, Vickers indenter, Ulm, Germany), the respective indentation devices were calibrated with respect to the instrument compliance standard [34]. The MIIT device is also equipped with an optical microscope that allows the simultaneous reading of Vickers indent diagonals after complete unloading. A peak load of 150 N was selected as a trade-off to fulfill the restriction on the narrow width of the deposit (Figure 2). However, other loads (25, 100, 400, 500, 700, and 1000 to 1200 N) were also investigated. In this study, only the 150 N load case (30 s holding time, 1 N/s loading, and 4 N/s unloading rates) was examined in detail for three macro-indents, on each of the X-Y and Y-Z planes, located at a sufficient depth along the deposit outline in the region below the hemispherical cap. This depth had to be consistent with the minimum distance between the indent and the surface (~3.5 d, where d is the diagonal of the Vickers indent), and the mean thickness of each deposited layer (~600 µm). For convenience, ten repeated MIIT cycles were performed at the same indent location, using the same peak load. MIIT analysis generally only utilizes indentation data deduced from the first cycle unless more detailed information is requested to clarify the elastoplastic behavior of the material. 

The hardness value obtained using the diagonal readings is defined as the equivalent macro Vickers hardness (HV_eq_) and can broadly be related with the standard macro Vickers hardness [39]. The hardness resulting from MIIT is hereinafter denoted with the term macro-indentation hardness (H_IT_). HV_eq_ differs from H_IT_ in the respective definition of the contact area, that is, the lateral contact area after complete unloading for the former, and the projected contact area on loading for the latter. Both definitions assume a perfect pyramidal geometry of the indent. Thus, HV_eq_ can be expressed in terms of H_IT_, provided the latter is corrected for an equivalent lateral contact area of the residual imprint (i.e., the 0.094 factor). This hardness is denoted hereinafter as *residual* indentation hardness (H_ITR_ in GPa).

The nIIT survey was performed at a maximum load of 5 mN (10 s holding time and 50 µN s^−1^ loading/unloading rate cycle). An extensive nIIT grid mapping survey was performed across various regions of the deposit, including its core, and on the X-Y plane (with its tip directed along the build direction, *Z*-axis). Nine representative nICs were selected at the center of the deposit, covering an area of 26 × 26 µm^2^, as representative of the deposit core grains (see Figure 2). It is worth noticing that nanoindentation test was applied to about 20 grains (selected at random) in the X-Y plane at the core of the deposit, and the results revealed that the mean values of the indentation modulus were in the range 160.8 ± 7.3 GPa likely depending on the orientation of the grains. However, some isolated grains exhibited even much larger or smaller values than 160.8 GPa. This result, although based on a small group of grains, indicates that the PBF-LB grains preferentially grow in the Y-Z plane along the <101> direction with some influence of the <100> direction. This grain anisotropy was confirmed by another study based on the EBSD of the same SS 316L alloy (see the results and discussion sections for details). For the sake of brevity, only the nanoindentation results within one single grain were reported in this work.

## 3. Results

**Figure 3:**
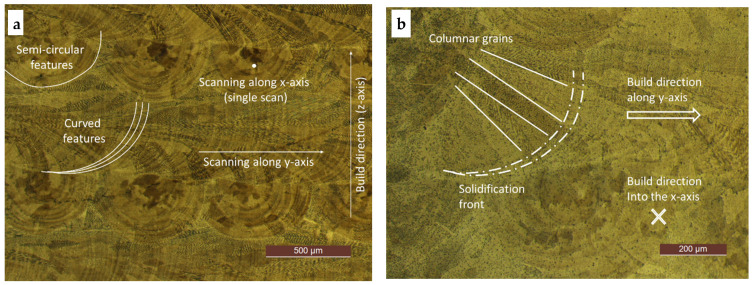

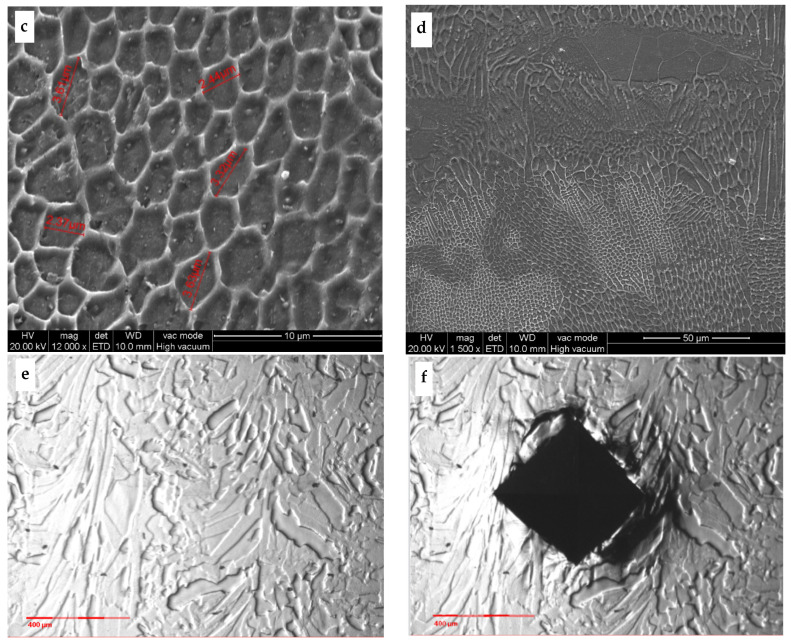
Microstructures from optical microscopy obtained after etching with aqua regia: (**a**) Etched micrograph at a magnification of 5× from OM revealing curved and semi-circular trace features; (**b**) Details of columnar grains at a magnification of 10×, with an embedded cellular structure; a narrow region along the solidification front appears relatively brighter, likely due to a greater content of passivating elements (Cr and Mo); (**c**,**d**) SEM images of the characteristic cellular structure of either an equiaxed or elongated morphology; (**e**,**f**) Unetched nano-polished PBF-LB 316L samples at a magnification of 5× before and after MIIT, respectively, with a peak load of 700 N in the core of the deposit and after nIIT (not shown).

The typical microstructure of the laser deposited SS 316L steel in the Y-Z plane is shown in Figure 3a,b, as observed using the optical microscope. These figures show two layers deposited in the building direction (*Z*-axis), with the two alternating scanning directions, namely, the X-direction and the Y-direction. The same figures show the melt-back phenomenon [40], which involves the simultaneous melting of the upper layer and re-solidification of the underlying layer, where the high local temperature causes the growth of equiaxed grains in the center of the circles (melt pool). The two perpendicular scanned directions determine two characteristic melt fronts, that is, overlapping curved and semi-circular, respectively. The large equiaxed grains are within layers with semi-circular traces (X-scanning direction), whereas the elongated grains are within layers with curved traces (Y-scanning direction). The mixed equiaxed and elongated cell structure was better resolved with SEM at a 1500×–12,000× magnification, albeit only within the curved trace layers (Figure 3c,d). The microstructure indented with the 700 N peak load is shown in Figure 3e,f. The same region has also been probed by means of nIIT, and the results are shown in Figure 2. As can be seen, the coarse grains are quite irregular and have a very high aspect ratio and a wide average size distribution (70 to 130 µm). 

With respect to the chemical homogeneity, some local changes were detected at the overlap between the curved traces, as shown by the brighter etching in Figure 3b. This might result from an enrichment of passivating elements, especially Cr and Mo, as shown in other studies [41]. It was also confirmed that the matrix was exclusively composed of austenite. Optical micrographs, taken after etching, revealed the occurrence of very sporadic micro-pores or submicron pores. These pores were not observed before etching and might be corrosion pits that occurred after multiple etching trials.

**Figure 4:**
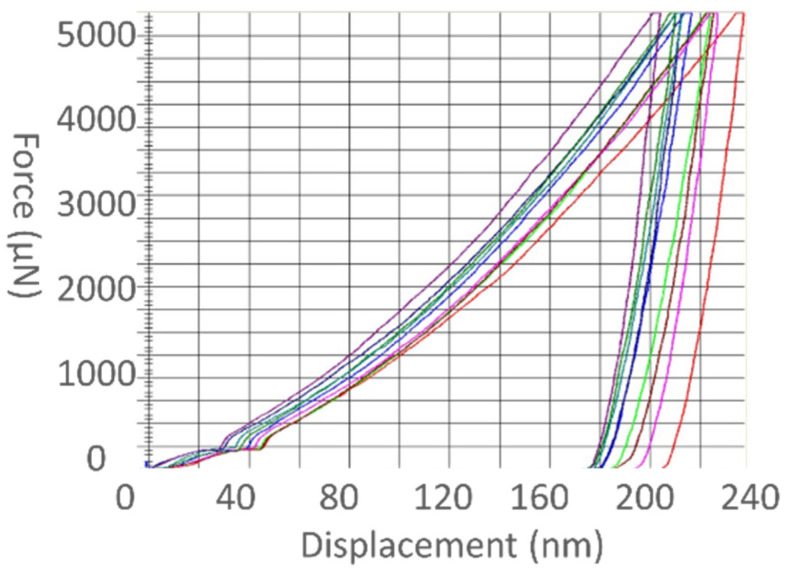
Nano-indentation curves (load control, 5 mN peak load) in the X-Y plane at the core of the deposit face.

The resulting indentation properties evidenced similar but apparently uncorrelated outcomes. The nIIT survey with a 5 mN peak load enabled us to sense the SS 316L steel microstructure inside the large single grains. A set of only nine nanoindentation curves (nICs), taken from an extensive grid mapping across the deposit, is depicted in Figure 4 for clarity reasons. The measured nICs all follow similar trends during loading, holding, and unloading. The maximum penetration depth is 240 nm on average. The loading curve of the analyzed nICs (Figure 4) shows common anomalies at a depth of ~40 nm. The average indentation modulus and hardness were estimated as 153.2 ± 4.7 and 3.2 ± 0.1 GPa, respectively (see Table 1 for the complete list of values). The nanoindentation hardness (nH_IT_) and modulus (nE_IT_) appear to be uncorrelated to each other. 

**Figure 5:**
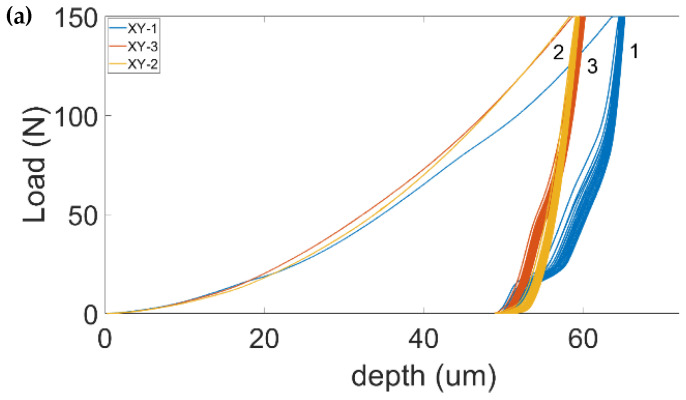

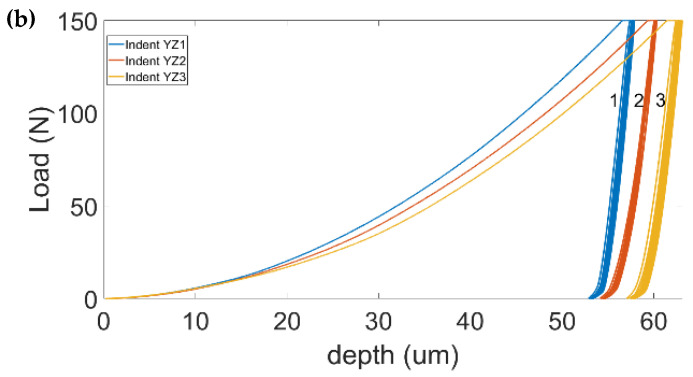
(**a**) Macro-ICs (150 N peak load) at corner locations of the deposit face in the X-Y plane. Although the loading of the three ICs is comparable, ICs 1 and 3 exhibit abnormal behavior, which was ascribed to an incorrect touching of the two indenter-sensors against the deposit during complete release of the load (see discussion in the text). (**b**) Macro-ICs (150 N peak load) at the core of the deposit face in the Y-Z plane.

Figure 5a,b depict the ten multicycle MIIT curves (ICs) in the X-Y and Y-Z planes of the deposit, respectively. The Vickers tip reached maximum penetration depths of 66.24 and 63.56 µm on average for the X-Y and Y-Z planes, respectively. The two ICs in Figure 5a (blue (1) and red (3) colored) in the X-Y plane exhibit an abnormal unloading behavior, as shown by the unusual presence of inflection points and steep changes in the slope. The yellow (2) one appears more regular, with a similar elastoplastic behavior and h_max_ to (3), but slightly different plastic depth, due to the irregular unloading of the latter. The loading and unloading behaviors of the three ICs in Figure 5b in the Y-Z plane is qualitatively comparable but exhibit different plastic and maximum depths and contact stiffness. The average maximum depth of the three curves in the Y-Z plane also compares well with the regular IC (2) in the X-Y plane. The measured macro-indentation hardness (H_IT_, third column in Table 2) are 1.74 ± 0.16 and 1.78 ± 0.12 GPa for the X-Y and Y-Z planes, respectively. The values of the indentation modulus (E_IT_, second column in Table 2) are 122.84 ± 10.15 and 174.38 ± 10.50 GPa for the X-Y and Y-Z planes, respectively. Thus, although the indentation hardness values of both planes compare well, even in terms of standard deviation, the indentation modulus appears to be quite different, even though the macro-imprint that covers several grains should minimize any existing effect of crystal anisotropy. The values corresponding to the equivalent Vickers (HV_eq_, fourth column) and the residual indentation hardness (H_ITR_, ninth column) along with the length of the two diagonals (d_h_, d_v_ in the fifth and sixth columns) taken from the residual imprint are also reported in Table 2. The trend of E_IT_ in the Y-Z plane (Table 2) is direct versus H_IT_ but inverse as regards HV_eq_ and H_ITR_. 

The ultimate tensile stress-like values in Table 1 and Table 2, estimated assuming the well-known reference properties approximation introduced by Tabor (~H_IT_/3), are within the 1072.6 ± 43.8 and 587.2 ± 48.8 MPa ranges, respectively, although the value attained from the first IC in the X-Y plane may underestimate the true values, as it is in contradiction with the more uniform values of HV_eq_ over the same X-Y planes of 179.78 and 191.58 kg_f_/mm^2^, although a somewhat lower range of 158.9−165 kg_f_/mm^2^ was found in the Y-Z plane, which, in turn, was in contradiction with the higher indentation hardness range (1.63−1.93 GPa) in the same Y-Z plane. These results suggest a lack of correlation between the equivalent Vickers hardness and the indentation hardness.

## 4. Discussion

### 4.1. Ante Fact: Correlation between Nano- and Macro-Indentation Properties

Laser fabrication methods can expose the initial powder to a very localized energy to promote rapid melting and solidification. The observed microstructure is due to very large thermal gradients [42] and variations in the cooling rate. The selected laser scanning strategy, which was based on alternating layers, produced a typical Y-Z plane microstructure after etching (Figure 3a,b) that was made of alternating stripes (of thickness of ≈60 and ≈50 µm) of overlapped semi-circular and curved melting fronts. Figure 3c,d show a cellular structure (average cell size of about 2 µm) embedded in much larger columnar grains, which are prominently observable in the curved solidification fronts that evolve along the *Y*-axis scanning direction. Much smaller cells (smaller than 2 µm) can also be observed near the outer surface of the deposit. The elongated grains are driven by the large thermal gradients that build up perpendicular to the advancing curved solidification front. Similar microstructure morphologies have been reported by other researchers for an identical PBF-LB SS 316L steel [43]. Additionally, typical equiaxed grains can be observed in the layers with a semi-circular solidification front advancing along the *X*-axis scanning direction. Overlapping curved layers cause grain growth underneath equiaxed grains within a semi-circular X-direction layer [44], and equiaxed and elongated grains were also observed in the top and side views when investigating the L-PBF fabricated 316L. An optical micrograph (Figure 3e,f) of the same microstructure in the Y-Z plane, without etching, reveals the characteristic dendric branching structure made of coarse grains with a relatively wide aspect ratio (10 to 100). 

The elastoplastic properties of such a microstructure are sensed, by nIIT, at the superficial single grain level (similar to a single crystal) at the core of the deposit and, by MIIT, across its bulky polycrystalline structure along the boundary of the deposit. 

The anomaly shown in the nICs for loading at very low loads (Figure 4) has been attributed to an initial elastic interaction between the indenter tip and the material (e.g., due to an oxide film) which gradually becomes unstable (slope change) and degenerates into an abnormal inelastic displacement under the same critical load. The Berkovich indenter is responsible for the essential elastoplastic behavior of the material above this critical load. We assumed, as discussed later, that such an initial anomaly did not influence the characteristic elastoplastic behavior of the material under larger loads. A striking feature of the nICs is their prominent large shift in the on-loading maximum penetration depths (204–235 nm) and, consequently, upon unloading. Table 1 shows that, in spite of the possible influence of ISE on nIIT [18,45], the measured nH_IT_ (3.2 ± 0.13) is nearly constant within the nanoscale probed region. This is consistent with the presence of a homogeneous solid solution in austenitic grains (nearly single crystals). The measured nIIT hardness values compare well with the 3.4 GPa reported by [46], when nIIT was performed, under depth control, at a maximum penetration of 800 nm (about four times the maximum depth of this study). Zhai et al. [47] reported an nIIT hardness value of 3.12 ± 0.14 GPa for a similar material while performing nIIT with 1000 nm of maximum depth (nearly five times the maximum depth considered in our study). Zeng et al. [48] performed an nIIT on a similarly fabricated SS 316L with a peak load of 50 mN (10 times higher than ours) and reported a nano-hardness value of 3.25 ± 5 GPa. However, these previously mentioned nH_IT_ values were much larger than the 1.95–2.03 GPa found by Khodabakhshi et al. [49] under a peak load of 200 mN (40 times greater than that used in the current study). 

As already noted, the measured indentation hardness and indentation modulus at the nano-scale seem to be uncorrelated to each other and to the measured macroscopic properties, very likely due to the intrinsic anisotropic properties of 316L in such large grains [27,46]. Hausild et al. [50] in fact showed a large anisotropy effect in a single crystal of conventional SS 316L (~120 GPa in (001), 200 GPa in (101) and up to 280 GPa in (111) planes. Moreover, they conducted a systematic investigation that addressed the nanoscale indentation properties of large (single crystal) grains of an SS 316L alloy produced by laser powder bed fusion, considering different grain orientations and build planes. They proved the significant influence of nanoindentation hardness and indentation modulus on the texture and orientation of the grains. It has been confirmed, in the present study, that relatively coarse grains in the PBF-LB 316L alloy behave as if they were single crystals, thereby exhibiting their own intrinsic elastic anisotropic properties. 

The undesirable effect of ISE clearly manifests itself when nH_IT_ is compared with H_IT_ values, as shown in Table 1 and Table 2. The measured values of H_it_ and E_it_ are 1.7 ± 0.1 and 117.7 ± 29.7 GPa, respectively. With reference to the work of [51], we noticed that the ISE-free measured nH_IT_ range compares well with our H_IT_ range rather than with our nH_IT_ one. The dispersion of H_IT_ and nH_IT_ values found here is equivalent, but the E_it_ values are much more dispersed than the nE_IT_ counterparts. This shows that H_it_ and E_it_ are somehow correlated, but nH_it_ and nE_it_ are not. As far as the macroscale is concerned, this behavior cannot be ascribed to ISE or to any eventual superficial artefacts, as these have less influence at large depths of the order of 60 µm. The equivalent Vickers hardness is in the 186.71 ± 5.03 range and 162.67 ± 2.87 HV_15_ range for the two planes and does not follow the trend of H_IT_. It is worth noting that, although H_IT_ is measured during loading/holding, Vickers hardness relies on residual indenting, i.e., after complete unloading. The measured equivalent Vickers hardness was compared against the literature values for the PBF-LB SS 316L steel, although the latter values refer to different process conditions. For instance, we found from [52] values of 220–225 HV_10_, Ref. [53] reported values of 223–245 HV_30_, Ref. [54] measured values of 215–255 HV, and Ref. [55] obtained values of 325 ± 15 HV_1_. All the above macro-Vickers ranges reported in the references are slightly larger than those found here. In this study, porosity and chemical composition were excluded as factors of primary influence, and it was considered that texture, microstructure, or some other important factors may have come into play. Regrettably, as macro-indentation testing is not so common in research, to the best of the authors’ knowledge, no useful macro-Vickers IIT data were found for the given 316L alloy that could be used for comparison purposes. Nevertheless, an indirect comparison could be made by comparing the estimated UTS-like values (581.1 ± 55.06 and 593.3 ± 40.8 MPa for the X-Y and Y-Z planes, respectively), which are listed in Table 2 with the UTS found by other researchers, that is, 595–635 [35], 509–668 [36], 540–630 [55], 511–698 [53], 555–690 [54], and 572.8 ± 0.6 [56] for the same as-built PBF-LB 316L alloy. This good match would seem to encourage the use of MIIT hardness to deduce representative tensile-like properties of polycrystalline PBF-LB products in the absence of more specific macro-Vickers IIT data that could be used for comparison purposes. However, the lower UTS value of 503 MPa estimated here may appear suspicious, particularly when associated with the relatively low values of H_IT_ and E_IT_. Such a sudden drop in UTS cannot be ascribed to microstructure heterogeneities or internal defects, as no significant chemical composition changes were detected with EDS and no marked porosity or inclusions were observed, even at a high magnification. 

A comparison between the measured E_IT_ and Young’s modulus was attempted. The typical values of a polycrystalline SS 316L alloy, produced by means of conventional manufacturing, are in the 180–190 GPa range. Kurzynowski et al. [37] and Chao et al. [56] reported the tensile properties of SS 316L produced by means of PBF-LB, before and after stress-relief heat treatments, including those of other authors. The reported Young’s modulus values were generally few in number and quite widespread, that is, 219 GPa by PBF-LB [37], 169–212 GPa after a stress relief heat treatment [37], 195 GPa by PBF-LB [57], and 150–193 by PBF-LB [58]. When these data are compared with the E_IT_ values shown in Table 2, they appear slightly larger than those in the Y-Z plane (174.38 ± 10.49 GPa), but markedly larger than those in the X-Y plane (122.84 ± 10.15 GPa). Since Young’s modulus of a polycrystalline material is a microstructure-insensitive property, the difference in E_IT_ in the two planes of the same alloy appears to be unsound; therefore, further investigations are required. 

A notable aspect of interest is the abnormal plastic deformation pattern that can be observed in Figure 3f around the residual macro-indents over the Y-Z plane, with a 700 N peak load. Figure 3e shows the same microstructure as Figure 3f, but before indentation. The indented area covers several coarse grains. The plastic strains seem to be effectively accommodated by the coarse grains, which are favorably oriented toward the top and bottom right edges, whereas the grains at the bottom-left edge are barely affected. A crack can be observed in the top-left edge, which is unusual for an SS 316L alloy, as it is expected to be ductile. Such a crack can be attributed to unforeseen tensile residual stresses (TRS). Although residual stresses of various types are quite common in strongly non-equilibrium PBF-LB builds [59,60,61,62], we presumed that the multiple slicing and polishing of our sample led to a complete relaxation of RS, and thus, it could be ignored in our study plan. 

Figure 3f, on the other hand, offers evident counterproof on the presence of TRS, which, among others, could explain the origin of anomalies on the measured indentation properties and their dispersion.

An attempt has been made in the next section to reassess the overall range of indentation properties considering the presence of RS in the PBF-LB SS 316L steel sample. This serves to rationalize the effect of RS on the measured indentation properties and ultimately verify their reliability.

A more in-depth inspection of the values listed in Table 2 has revealed further anomalies, the most relevant being the much smaller diagonal lengths in the X-Y plane than those in the Y-Z plane (390 vs. 415 µm), in spite of the identical peak load used for both planes.

Moreover, the trend of the equivalent Vickers hardness (HV_eq_) does not follow that of H_IT_, although a distinction should be made between the former and the latter based on their respective definitions. Indeed, the former is a pure plasticity index that is determined from the mean value of the two measured Vickers diagonals (fifth and sixth columns) over the residual imprint. The latter is instead a lumped elastoplastic index that is determined by using an area function (derived from a priori calibration of the indenter against a standard reference material), which is computed at the attained maximum penetration depth.

When RRS is present across the sample, these two indices sense the material in different ways. The residual imprint, linked with HV_eq_, is determined by the ultimate inelastic response of the material including some relaxation of the initial RRS. Conversely, H_IT_ senses the direct interplay between the (elastic) RRS and the elastoplastic indentation phenomena underneath the indenter. From this perspective, the two hardness indices become lumping parameters, which, in addition to conventional contributions, also include different effects of the RRS. 

It is interesting to see, in Table 2, that the largest maximum penetration depth is always consistent with the lowest H_IT_ in both the X-Y and Y-Z planes, thereby confirming an acceptable constancy of these parameters (including a UTS-like parameter) versus RRS. However, H_IT_ is often uncorrelated with HV_eq_ in both planes. Some indents in either plane may occasionally exhibit a significant difference in diagonal lengths (equal to or larger than 12 µm). Such a general mismatch in indentation data is fully reflected on the anomalous values of E_IT_ from all the indents, and this is why it is believed to be the main indicator of RRS in the sample. This examination of Table 2 also suggests that the main difference in the indentation parameters between the two planes and from the macro-indents should not be attributed to a different microstructure morphology, heterogeneities, or anisotropy, but instead to a distinct role played by RRS.

### 4.2. Post Fact: Influence of Residual Stress on Macro-Indentation Properties

The study of RS by means of IIT is a subject of current active research [62,63]. Although several methods have been proposed, none of them is free of application difficulties [62,63]. Indeed, many of them require the a priori determination of a zero-stress reference state, which is often unknown [62,63]. Moreover, their experimental validation, e.g., by means of X-ray diffraction or similar, is very difficult at the greatest depth of interest in this study (60 μm). Thus, although the main goal of this study has been to determine the tensile-like properties of the PBF-LB 316L alloy by means of MIIT, another goal has been to determine whether the measured indentation properties are true or RS-invalidated ones. With the RS as a new playing factor, the unloading anomalies shown in Figure 5a could also be a consequence of free distortion of the sample upon RS relaxation during MIIT unloading rather than an unlike accidental sample preparation.

Before analyzing the new indentation data, the issue of RS in metals and alloys is briefly introduced, the most salient results attained so far are summarized, and the new experimental strategy is presented along with the necessary working assumptions. 

The experimental IIT results discussed in the previous section at a nanoscale and macroscale comply with the ISO 14577-1,2 procedure, which assumes the presence of a pure elastic contact at the interface between the indenter surface and the material upon incipient unloading. This implicitly implies the absence of any RS in the material. Moreover, as RS may induce sink-in and pile-up phenomena, these phenomena are not covered by the above code either. Thus, with the presence of RS in the PBF-LB fabricated SS 316L alloy, the invoked standard procedure may provide inaccurate results. 

Chen et al. [44] classified residual stresses into three categories: type I, which is at the macroscale and is caused by plastic deformation gradients, and type II and III, which are found at the microscale and atomic levels. Type II is associated with the grains in an intergranular manner owing to the strain incompatibility among the grains, while type III is associated with the grains in an intragranular manner, with dislocated cells within a grain, respectively. Accordingly [44,64], type I residual stresses are pertinent to this study and can be measured with MIIT. Expectedly, owing to prior slicing and polishing operations, the original RS imparted by the PBF-LB process to the investigated deposit had been relaxed to some extent, and hence, denoted hereinafter as remnant residual stress-strain field (RRS).

Tsui et al. [65] simulated a standard indentation process by means of the finite element method and proved that the indentation hardness and indentation modulus increase as the compressive residual stresses increase, but decrease as the tensile residual stresses increase. However, our fundamental knowledge on Young’s modulus of materials indicates that it should remain approximately unaffected by changes in the microstructures with any fabrication method. 

The indentation data listed in Table 2 are now complemented with those derived from repeated macro-Vickers IIT indentations (under a constant peak load, but the same indent) to better elucidate the role of RS and its relaxation behavior upon indentation of the PBF-LB 316L alloy. Previous researchers successfully used repeated macro-Vickers IIT indentations to improve the measurement accuracy of the indentation modulus in various metals, especially those with a low strain hardening ability [66]. 

The difference between the indentation properties over the two planes is expected to be due to different types of RS originated by the PBF-LB process and the different cuts performed in the original deposit. For simplicity, we assume that the RRS are purely elastic. A larger or lower value of E_IT_ than the Young’s modulus will provide an index of the sign (i.e., compressive or tensile, respectively) of the RRS [64].

The E_IT_ values listed in Table 2 for the X-Y plane are much lower than those in the Y-Z plane. Both planes also show lower E_IT_ values than Young’s modulus. We initially ascribe this discrepancy to the influence of the tensile RRS present in the PBF-LB 316L alloy sample [65]. The presence of tensile RRS was reported in PBF-LB fabricated 316L alloy [67]. These were typically caused by large thermal gradients or large cooling rates [37]. Tensile RRS alter the measured indentation curves (IC) causing a marked shift to the right of the loading segment of the IC and a shift to the left of the unloading segment of the IC [62,63]. 

As H_IT_ is closely linked to residual strains, as a first approximation, it can be considered insensitive to residual stresses. The consistency of H_IT_ across the sample can be appreciated from the matching observed between the estimated UTS-like parameter (≈H_IT_/3) and the UTS values of the PBF-LB 316L alloy in the literature. As H_IT_ depends on the maximum penetration depth (h_M_), it may be a useful parameter to sense in-depth RRS. In this study, an alternative hardness index, that is, HV_eq_, has been introduced.

HV_eq_ resembles the standard HV in that it utilizes the same procedure to determine the residual lateral contact area. However, HV_eq_ differs from the standard HV as it employs an MIIT cycle rather than a standard HV cycle.

In this work, the H_IT_ and HV_eq_ indices should be interpreted in a different light when associated with RRS. The main difference between the standard H_IT_ and HV_eq_ concerns the contact area, that is, the projected contact area for H_IT_ and the lateral contact area for HV_eq_. Their major difference lies in the indentation cycle. The former is determined upon loading/holding, and typically senses the elastoplastic properties of the material. The latter is determined after complete unloading, and hence, represents a pure plasticity index of the material. However, when RRS are present in the sample, the two indices provide quite different outcomes. In the case of H_IT_, competition occurs between the elastoplastic field induced by the indenter and the interfering elastic RRS. Thus, H_IT_ measures the combined effect of both phenomena. Even though we assume an identical loading–holding cycle for HV_eq_ to that used for H_IT_, upon complete release of the indentation load, the detected lateral contact area of the residual imprint now accounts for the combined effects of indentation hardening, its eventual elastic relaxation, and the relaxation of RRS (if any). The conventional meaning of HV_eq_ may be modified to a great extent, depending on the relative importance of the initial tensile RRS on the indentation plasticity phenomena. If tensile RRS are dominant, large in-plane permanent displacements are accompanied by incomplete strain hardening, which results in a misleading HV_eq_. Alternatively, if the initial RRS can be substantially relaxed during indentation, thereby allowing sufficient indentation strain hardening, the HV_eq_ results to be a conventional hardness index. Moreover, both the standard HV and HV_eq_ can mask the presence of large tensile RRS, and caution should therefore be taken when using the mean value of the two Vickers diagonals, especially when a large difference in the diagonals is detected.

The difference in the residual Vickers diagonals is considered as an indicator of in-plane two-dimensional tensile RRS. Although compressive RRS are expected to induce relatively small diagonal differences, tensile RRS are responsible for much larger values. Furthermore, small diagonal differences do not necessarily imply negligible RRS. For instance, compressive RRS in one direction may relate to tensile RRS in the orthogonal direction and confine the net deformation underneath the indenter. Alternatively, the indenter diagonals may accidentally be aligned along the RRS-balanced directions, thereby resulting in negligible diagonal differences. 

Table 2 shows that the diagonal differences measured in both the X-Y and Y-Z planes range between 12 and 15 μm, which means that both planes are affected by tensile RRS, as also attested by the low values of E_IT_ at all the indents_._

**Figure 6:**
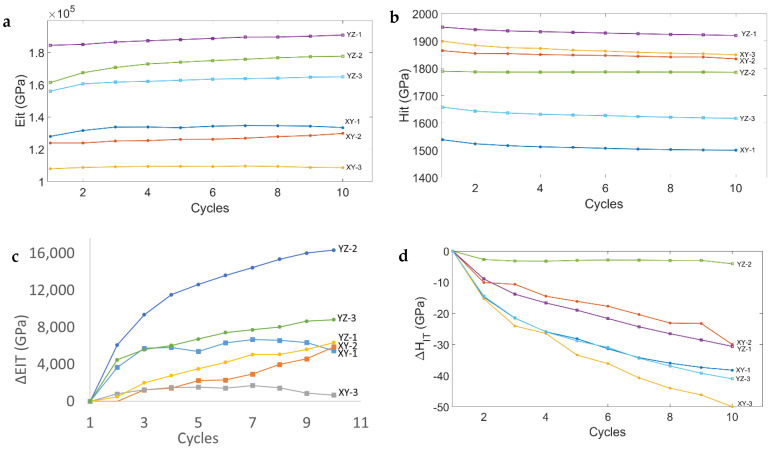
Multi-cycle (10 cycles) plots of (**a**) EIT for each cycle on the X−Y and Y−Z planes, (**b**) HIT for each cycle of the six indents on the X−Y and Y−Z planes, (**c**) the progressive increase in EIT, with respect to the increase in the first cycle (diff-EIT), and (**d**) the progressive decrease in HIT with respect to the decrease in the first cycle (diff-HIT).

With reference to the repeated indentations, the changes in E_IT_ and H_IT_ at the nth indentation cycle are computed with respect to the first cycle. The changes in indentation modulus and indentation hardness are computed as: E_it_(*n*) − E_it_(1) = Δ*E_it_*(*n*), and H_it_(*n*) − H_it_(1) = Δ*H_it_*(*n*), respectively. Figure 6c and Figure 6d show such changes in the two indentation properties after ten cycles [14,34]. 

In the absence of RRS, repeated Vickers indentations promote a fully developed plasticity condition to be attained (also called Tabor’s condition), which is reached upon IIT loading [66], especially in metals and alloys that exhibit a poor strain hardening ability. In the presence of RRS, repeated Vickers indentations are expected to relax RRS to some extent via the plasticity developed underneath the indenter. Table 2 shows the two measured diagonals of the residual Vickers imprint (5th and 6th columns) over the X-Y and Y-Z surfaces. 

The results listed in Table 2 and the ones from repeated Vickers indentations over the X-Y and Y-Z planes are discussed hereafter.

**Figure 7:**
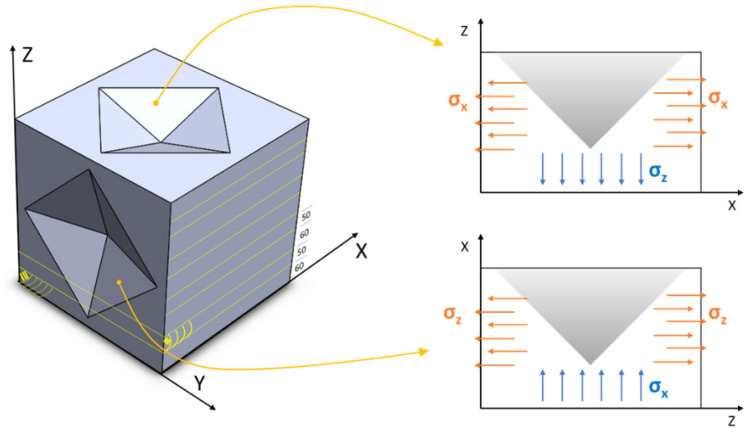
Three-dimensional schematic illustration of in-plane and in-depth residual stresses with reference to the macro-indents in XY and YZ planes (which are highlighted in Figure 2) and detail of indent 2D cross section in both planes underlining the effect of residual stress.

It should be recalled that the deposit is relatively thin along the y-direction, and that the deposition vector along z is much smaller than that along the x direction; thus, in-plane σ_y_ (residual stress) (see Figure 7) is assumed to be negligible in comparison to σ_x_ and σ_z_; moreover, alternate overlying Y-deposit layers tend to further relax the area underneath σ_y_ as a result of tempering.

The two opposed lateral surfaces of the deposit are likely to induce tensile RRS, due to rapid cooling. The thickness of the deposited layer along the x-direction (X-layer) is ≈60 μm, whereas the thickness of the deposited layer along the y-direction (Y-layer) is ≈50 μm. These two thicknesses have the same order of magnitude of the maximum penetration depth (≈60 μm) reached during MIIT with 150 N peak load. HV_eq_ provides an index of the unrelaxed tensile RRS upon IIT, and thus, can measure the extent to which an indentation property is affected by RRS. 

The three macro-indents lying over the X-Y plane located a large distance from the substrate–deposit interface. This distance ensures: (i) the same in-depth σ_z_ acts at the three indents, (ii) any chemical inhomogeneity (e.g., due to dilution between the filler and the substrate) is negligible, and (iii) the thermal condition of each indent mainly depends on the local cooling rate (CR). 

The active residual stresses over the X-Y plane are in-plane σ_x_ and in-depth σ_z_. The X-Y plane experiences large in-plane tensile σ_x_ and constant σ_z_.

Indent **#1**, X-Y plane:

- **location**: near the outer lateral surface of the deposit, and near the top dome.
- **RRS**: large tensile σ_x_ resulting from a high cooling rate (CR) on the outer lateral surface and large tensile σ_z_ affected by the high CR at the top dome.
- **properties**: the large tensile stresses are consistent with the very low value of the indentation modulus (much smaller than Young’s modulus). The low value of H_IT_ and UTS-like is linked to the large value of h_M_; the small value of the diagonal difference (Δd) does not offer any helpful information; the HV_eq_ value in Table 2 suggests a relatively large value of unrelaxed RRS.
- **outcome**: softest location, unreliable indentation properties due to strong RRS.

Indent #2, X-Y plane:

- **location**: farther than #1 from the lateral outer surface and near the top dome.
- **RRS**: milder CR from the lateral surface, and identical large tensile σ_z_ to #1, due to the high CR from the top dome.
- **properties**: although the HIT of this indent coincides approximately with that of #3, HV_eq_ is lower than that of #1 and #3, which means lower tensile σ_x_ than in #1 and #3, and slightly more reliable indentation properties than those of #1 and #3. E_IT_ is still very low; the large value of Δd clearly confirms the presence of tensile RRS.
- **outcome**: despite the strong presence of high unrelaxed tensile RRS, the HV_eq_ value suggests a less invalidating influence on the indentation properties at this indentation than the other two.

Indent #3, X-Y plane:

- **location**: similar to location of #1, that is, near the other surface and close to the top dome, as #1 and #2.
- **RRS**: a similar CR effect of #1 from the lateral surface and from the top dome; identical large tensile σ_x_ and σ_z,_ as in #1; the large value of Δd clearly confirms the presence of unrelaxed tensile RRS.
- **properties**: the largest HV_eq_ value suggests a strong invalidating effect of unrelaxed RRS. The H_IT_ and h_M_ values of #3 are approximately identical to those of #2, but inconsistent with the larger UTS-like and lower Δd values of #3 than #2; moreover, the low value of E_IT_ proves the low reliability of the indentation properties of indentation #3.

The active residual stresses over the Y-Z plane are in-plane σ_z_ and in-depth σ_x_. The Y-Z plane exhibits a more complex stress state than the X-Y plane.

Indent #1, Y-Z plane:

- **location**: at the core of the deposit, near the top dome.
- **RRS and properties**: the large value of Δd suggests the presence of tensile σ_x_; on the other hand, the large H_IT_ and UTS-like values indicate an effective synergism of compressive σ_z_ with tensile σ_x,_ whereby the small h_M_ value is also attained. This is consistent with a location at the inner core of the deposit, conceivably under high compressive σ_z,_ which diminishes the detrimental effects of tensile σ_x_. The synergistic contribution of tensile–compressive RRS is consistent with the value of E_IT_, which here is almost identical to Young’s modulus of the alloy. This is clear evidence that an E_IT_ nearly equal to Young’s modulus does not necessarily mean nearly zero-RRS; moreover, the lowest HV_eq_ value is attained, thus indicating the minimal influence of RRS.
- **outcome**: the strongest and the hardest location of the tested deposit with the most reliable indentation properties.

Indent #2, Y-Z plane:

- **location**: near the substrate–deposit interface, at the inner core of the deposit.
- **RRS and properties**: the small value of Δd is not helpful; the value of h_M_ compares well with that of # 2 in the X-Y plane, and the acceptable H_IT_ and UTS-like values, which are lower than those of #2 in the X-Y plane, also suggest a beneficial synergism of compressive σ_z_ (though lower than #1) with tensile σ_x_, as shown by the relatively low values of HV_eq_ and the core location of the indent. The reduced compressive stress could be attributed to the closer vicinity of the indent to the substrate, which induces slightly larger tensile σ_x_, due to thermal gradients. Although E_IT_ is smaller than that of #1, it is still close to Young’s modulus of the alloy, thereby indicating nearly reliable indentation properties.
- **outcome**: nearly reliable indentation properties.

Indent #3, Y-Z plane:

- **location**: near the outer later surface, at the mid-height of the deposit.
- **RRS and properties**: the effect of the substrate-induced thermal gradient on tensile σ_x_ has less influence here that in #2; however, the vicinity to the outer surface enhances the CR effect and the build up of larger tensile σ_x_, which are more difficult to relax during indentation. The value of Δd is not helpful. The value of h_M_ is larger than that observed in #2. The synergistic effect introduced by compressive σ_z_ is dramatically reduced, as shown by the relatively lower H_IT_ and UTS-like values, whereas HV_eq_ remains identical to that of #2; nevertheless, E_IT_ is smaller than that in #2, thus indicating a loss of reliability of the indentation properties.
- **outcome**: less reliable indentation properties than #2.

The repeated indentation test highlighted that indents affected by pure tensile RRS exhibit a faster increase in E_IT_ during the initial cycling than when a steady state is reached after completion of the cycle; this suggests that tensile RRS are more difficult to relax after indentation. Conversely, where compressive RRS are present, even in combination with tensile RRS, a linear increase in the indentation modulus corresponds to an effective relaxation of RRS, as has been observed for #1 in the Y-Z plane. It should be noted that although E_IT_ increases linearly after indentation #2 in the X-Y plane, as in the case of #1 in the Y-Z plane, the severe tensile nature of RRS does not allow their effective relaxation.

## 5. Conclusions

A defect-free, chemically homogeneous PBF-LB fabricated AISI 316L steel alloy has been investigated by means of the macro- and nano-scale instrumented indentation test (IIT) over the X-Y and Y-Z planes after conventional sectioning and metallographic preparation of the samples. The microstructure was composed of equiaxed grains and relatively coarse irregular (elongated) grains, which were embedded in a typical cellular-dendritic substructure. 

Nanoindentation testing over the X-Y plane showed that the grains were nearly stress free and had very little in common with the indentation properties at the macroscale. The measured indentation modulus was unaffected by the grain boundaries, while the characteristic intrinsic elastic anisotropy of the individual grains was revealed. The nanoindentation hardness did not vary to any great extent across the individual grains, although they suffered from a large ISE. 

Macro-indentation imprints over a few large grains in the Y-Z (growth) and X-Y (scanning) planes of the PBF-LB deposit showed that indentation hardness is affected less by ISE and provides a reliable estimate of the UTS-like (≈H_IT_/3) of the SS 316L.

Abnormal values of other indentation properties over the macroscale, such as the equivalent Vickers hardness and indentation modulus, indicated the need to reexamine the overall indentation results by considering the influence of residual stresses in the deposit. The presence of residual stresses in the indented sample implied a lack of justification of the basic requirements contained in the current ISO 14577-1 code, and therefore, that the appropriate meanings and outcomes of the traditional material parameters needed reconsideration.

It was found that H_IT_ provided a useful index that could be used to sense in-depth residual stresses, whereas the difference in the residual Vickers diagonals (whenever available) could be used to sense the in-plane stresses. Compressive residual stresses, even when combined with tensile stresses, were more effective in relaxing upon indentation and tended to ensure more reliable indentation properties; conversely, tensile stresses were found to be less effective in relaxing and more prone to invaliding the indentation results.

The developed methodology permits the influence of residual stresses on the measured indentation properties over the macroscale to be rationalized and the reliability of the measured indentation properties to be systematically discriminated.

## Figures and Tables

**Table 1:** Mean values of indentation hardness and indentation modulus measured by means of nIIT over an array of nine indents in the X-Y plane; UTS-like parameter (≈H_IT_/3).

Sr.	Peak Load	nHIT (Gpa)	nEIT (Gpa)	nUTS-Like (Mpa)
1	5 mN	3.4	156	1133.3
2		3.2	161	1066.7
3		3.2	159	1070
4		3.3	147	1126.7
5		3.3	155	1100
6		3.1	148	1043.3
7		2.9	149	980
8		3.1	150	1053.3
9		3.2	154	1080

**Table 2:** Averaged values of the indentation hardness, indentation modulus, Vickers hardness, and diagonals measured by means of MIIT (150 N peak load) over ten repeated cycles (except for hmax, which represents the maximum value at the 10th cycle). UTS-like parameter (≈H_IT_/3).

Ind.	E_IT_ (GPa)	H_IT_ (GPa)	HV_eq_	d_h_ (um)	d_v_ (um)	h_max_ (um)	UTS-Like (MPa)
X-Y plane							
1	133.14	1.51	188.77	388.69	386.48	66.24	503.3
2	126.35	1.85	179.78	389.65	404.65	60.72	616.7
3	109.02	1.87	191.58	378.78	390.69	60.68	623.3
Y-Z plane							
1	187.93	1.93	158.9	428.8	416.1	58.04	643.3
2	172.84	1.78	165	412.8	416	60.67	593.3
3	162.36	1.63	165	412.8	416.5	63.56	543.3

## Data Availability

The data presented in this study are available in article.
